# GLP-1R Agonist Exendin-4 Protects Against Hemorrhagic Transformation Induced by rtPA After Ischemic Stroke via the Wnt/β-Catenin Signaling Pathway

**DOI:** 10.1007/s12035-022-02811-9

**Published:** 2022-03-31

**Authors:** Chengli Liu, Shanshan Sun, Jie Xie, Hui Li, Tianyu Li, Qiqi Wu, Yongsheng Zhang, Xiangjun Bai, Jian Wang, Xin Wang, Zhanfei Li, Wei Wang

**Affiliations:** 1 Department of Traumatic Surgery, Tongji Hospital, Tongji Medical College, Huazhong University of Science and Technology, Wuhan, 430030 People’s Republic of China; 2 Department of Ultrasound Imaging Department, Tongji Hospital, Tongji Medical College, Huazhong University of Science and Technology, Wuhan, 430030 People’s Republic of China; 3 Department of Anatomy, College of Basic Medical Sciences, Zhengzhou University, Henan, 450000 People’s Republic of China; 4 Department of Neurosurgery, Brigham and Women’s Hospital, Harvard Medical School, Boston, MA USA

**Keywords:** GLP-1R agonist, Wnt/β-catenin signaling pathway, Tissue plasminogen activator, Ischemic stroke, Hemorrhagic transformation, Blood–brain barrier

## Abstract

Tissue plasminogen activator (tPA) is recommended by the FDA to dissolve intravascular clots after acute ischemic stroke (AIS). However, it may contribute to hemorrhagic transformation (HT). The Wnt/β-catenin signaling pathway plays an important role in regulating the blood–brain barrier (BBB) formation in the central nervous system. We explored whether glucagon-like peptide-1 receptor (GLP-1R) agonist exendin-4 (EX-4) reduces the risk of HT after rtPA treatment via the Wnt/β-catenin pathway by using a rat transient middle cerebral artery occlusion (MCAO) model *in vivo* and an oxygen–glucose deprivation plus reoxygenation (OGD/R) model in vitro. Our results showed that EX-4 attenuated neurological deficits, brain edema, infarct volume, BBB disruption, and rtPA-induced HT in ischemic stroke. EX-4 suppressed the production of ROS and the activation of MMP-9 to protect the integrity of the BBB by activating the Wnt/β-catenin signaling pathway. PRI-724, a selective inhibitor of β-catenin, was able to reverse the therapeutic effect of EX-4 in vivo and in vitro. Therefore, our results indicate that the GLP-1R agonist may be a potential therapeutic agent to decrease the risk of rtPA-induced HT after ischemic stroke via the Wnt/β-catenin signaling pathway.

## Background

The recombinant tissue-type plasminogen activator (rtPA) is the only drug approved by the Food and Drug Administration for the treatment of ischemic stroke [1, 2]. Nevertheless, the application of rtPA is restrained by its short therapeutic window and some severe adverse events. Hemorrhagic transformation (HT), as a common complication of rtPA thrombolysis, is mainly attributed to the direct aggravation of blood–brain barrier (BBB) breakdown [3]. The BBB is composed of cerebral endothelial cells, basement membrane, pericytes, and astrocytes [4, 5]. The neurotoxicity of rtPA involves the activation of matrix metalloproteinases (MMPs), the production of reactive oxygen species (ROS), and neuroinflammation leading to increased permeability and disruption of the BBB [6–8]. The occurrence of HT can aggravate the outcome of rtPA treatment in patients with acute ischemic stroke (AIS). Therefore, to increase the clinical application of rtPA and improve the prognosis of patients with AIS, it is of great clinical significance to develop strategies to protect the BBB and reduce the damage caused by HT.

Exendin-4 (EX-4) is an agonist of the glucagon-like peptide-1 receptor (GLP-1R) with effects on various physiological functions. Namely, EX-4 inhibits glucagon secretion and apoptosis. As a relatively small molecule, EX-4 diffuses across the BBB to directly access the central nervous system [9]. It has been reported that EX-4 exerts neuroprotective effects against oxidative, inflammatory, and apoptotic damage in stroke [10–12]. However, it remains unknown whether EX-4 reduces the risk of rtPA-reduced HT after ischemic stroke.

The canonical Wnt/β-catenin pathway plays an important role in the BBB formation via β-catenin stabilization [5, 13]. This facilitates the translocation of β-catenin to the nucleus, in which it binds to the transcription factors of the lymphoid enhancer (LEF)/T-cytokine family (TCF) to regulate gene transcription [14]. The serine–threonine kinase glycogen synthase kinase-3β (GSK-3β), which is a component of the adenomatous polyposis coli/axin/GSK-3β complex, plays an important role in regulation of the phosphorylation and degradation of β-catenin [15]. The inhibition of the Wnt/β-catenin signaling pathway may lead to damage of the BBB [16, 17]. Nevertheless, the role of the Wnt/β-catenin pathway in HT after rtPA thrombolysis in AIS remains unknown.

In this study, the middle cerebral artery occlusion (MCAO) rat model and the oxygen–glucose deprivation plus reoxygenation (OGD/R) model of cerebral microvascular endothelial cells (BMECs) were used to study the role of the Wnt/β-catenin pathway in tPA-induced HT by using EX-4. Meanwhile, the Wnt/β-catenin pathway was inhibited by PRI-724, a selective inhibitor of β-catenin, to further study the mechanism of EX-4. We hypothesized that the administration of EX-4 as a therapeutic drug would reduce the BBB permeability and the risk of HT and improve neurofunctional prognosis through the Wnt/β-catenin signaling pathway.

## Material and Methods

### Animals

Adult male Sprague–Dawley (SD) rats weighing 250–280 g were included in this study. The rats were raised in the Experimental Animal Center of Tongji Hospital and kept at 22 °C under standard conditions of 50–60% relative humidity and 12:12-h light/dark cycle. Standard food and water were supplied ad libitum. All animal experiments were approved by the Experimental Animal Ethical Committee of Tongji Hospital affiliated with the Huazhong University of Science and Technology.

### Transient Focal Cerebral Ischemia

Focal cerebral ischemia was performed by MCAO according to a previously described protocol [16, 18]. In brief, an intraperitoneal injection of 4% pentobarbital sodium (50 mg/kg) was used to anesthetize the rats. After making a midline incision in the neck, the left common carotid artery and external carotid artery were separated under a microscope and ligated temporarily with a 4–0 silk suture, and the internal carotid artery was clamped. An arteriotomy was performed proximal in bifurcation of the common carotid artery. A monofilament (40 mm long, 0.26 mm in diameter, Beijing Sunbio Biotech, China) coated with silicon nylon was introduced through the incision of the artery and entered forward into the internal carotid artery 18–20 mm, thereby occluding the beginning of the middle cerebral artery. Four hours later, rats were anesthetized again and the nylon monofilament was withdrawn to restore the blood flow of the middle cerebral artery. After the operation, rats were sent back to their own cages and housed in SPF units.

### Experimental Design and Drugs

Rats were randomly divided into five groups: sham group rats were treated with the same surgical operation without filament insertion, and they were administered 1 mL 1% DMSO intraperitoneally with 1 mL saline intravenously; vehicle group rats were treated with MCAO and administered 1 mL 1% DMSO intraperitoneally with 1 mL saline intravenously; rtPA group rats were treated with MCAO and administered 1 mL rtPA (10 mg/kg, Actilyse, Boehringer-Ingelheim, Germany) inserted into the left femoral vein 4 h after MCAO; rtPA + EX-4 group rats were treated with MCAO and administered EX-4 (100 µg/kg, MC Express, USA) intravenously immediately after rtPA injection; and rtPA + EX-4 + PRI-724 group rats were treated with MCAO and administered EX-4 intravenously and PRI-724 (10 mg/kg, Selleck, USA), a selective inhibitor of the β-catenin, intraperitoneally after rtPA injection.

### Neurologic Deficit Score

An investigator blinded to the experimental groups tested the neurological function in the animals 24 h after MCAO using a modified scoring system as described previously [16]. Each animal was scored according to following standards: 0, no symptoms of neurological deficits; 1, inability to completely extend the contralateral forelimb; 2, inability to straighten the contralateral forelimb; 3, mild circling to the contralateral side; 4, severe circling; and 5, no spontaneous activity, falling to the contralateral side. Higher neurologic deficit scores implied more severe injury of neurological function. Rats with subarachnoid hemorrhage and no neurological function were excluded.

### Brain Water Content

Brain edema was assessed by measuring brain water content with the wet–dry method as previously reported [19, 20]. The rats were anesthetized and sacrificed 24 h after MCAO. The brains were quickly removed, and the olfactory bulbs, cerebellum, and brain stem were discarded. The brain was placed on a dry surface and divided into the ipsilateral and contralateral hemispheres. Both hemispheres were wrapped in pre-weighed aluminum foil using an electronic analytic balance to obtain the total wet weight. After incubating at 100 °C for 24 h, the total dry weight was reweighed. The brain water content was expressed as follows: (total wet weight − total dry weight) / tissue wet weight × 100%.

### Measurement of Infarct Volume

The infarct volume was evaluated by the 2,3,5-triphe-nyltetrazolium chloride (TTC, Sigma-Aldrich) staining technique 24 h after MCAO, as previously reported [21, 22]. The animals were sacrificed, and the brain tissue was cut into seven coronal sections (2 mm thick) which subsequently stained with a 1% TTC at 37 °C for 30 min, and then fixed in 4% paraformaldehyde for 24 h. The photograph of stained sections was taken, and the digital images were measured using ImageJ image-processing software to calculate the infarct volume. The nonischemic tissue was stained red, whereas the infarct area remained unstained (white or pale). The ratio of infarct volume was expressed as the infarct area of the ipsilateral hemisphere divided by the total area of the ipsilateral hemisphere (× 100%).

### Evaluation of BBB Permeability

Evans blue (EB, 2%, 3 mL/kg, Sigma-Aldrich) dye was administered through the femoral vein 2 h before cardiac perfusion [4, 23]. After the brain was harvested, each hemisphere was weighed and homogenized in 4 mL of 50% trichloroacetic acid solution. Then, the homogenate was centrifuged at 12,000 rpm for 30 min, and the supernatant was collected and diluted with 100% ethanol at 1:3. The amount of EB dye was detected using a spectrophotometer at 620 nm and calculated by EB standard curve. The EB extravasation index was expressed as the ratio of absorption intensity of ischemic hemisphere to that of nonischemic hemisphere.

### Measurement of Myeloperoxidase

The level of neutrophil infiltration in the brain tissue was measured using the measurement of myeloperoxidase (MPO) assay kit (Jiancheng Bioengineering Institute, China) according to the manufacturer’s protocol [24]. The absorbance at 460 nm was assessed with a plate reader spectrophotometer. The MPO activity was calculated for each rat as follows: MPO activity (U/g tissue wet weight) = measured OD value − sham OD value / [11.3 × sample volume (g)].

### Spectrophotometric Assay of Hemoglobin Content

The concentration of hemoglobin in the ipsilateral brain tissue was assessed with a QuantiChrom hemoglobin assay kit (BioAssay Systems, CA, USA) as reported previously [4]. The rats were sacrificed, and immediately cardiac perfusion was performed. The brains were cut into the ipsilateral and contralateral hemispheres. After 10 mL/g of saline was added to each sample, the brains were fully homogenized and centrifuged at 13,000 rpm for 30 min. A 200-μL volume of reagent was then added to 50 μL of the supernatant. After 15 min of incubation at room temperature, optical density was measured at 400 nm with a spectrophotometer. A standard curve was acquired using the calibrator reagent in serial dilutions (0, 2.5, 5, 10, 20, 30, 50, 75, and 100 mg/dL).

### Hematoxylin–Eosin Staining

The coronal brain tissues of rats in five groups were stained with hematoxylin–eosin (HE) (C0105, Beyotime, CA) as reported previously [25, 26]. Briefly, paraffin coronal sections were dewaxed with xylene, rehydrated with gradient alcohol, and stained with HE reagent at room temperature for 11 min, then they were dehydrated with gradient alcohol and sealed. Finally, the cerebral histopathological images in the five groups were observed and photographed under a microscope.

### Immunohistochemical Staining

Based on our established protocol, the slices were incubated for 30 min at room temperature with 0.25% Triton X-100 in 1% BSA and for another 1 h in 5% BSA. Next, the slices were incubated with primary antibodies (anti-β-catenin [1:300, Abcam]) overnight at 4 °C, followed by incubation with biotin-labeled anti-rabbit IgG (1:500, Molecular Probes Inc.) for 1 h at room temperature. Then, an HRP-conjugated *Streptomycetes* working solution was added and incubated for 20 min. Finally, the slices were incubated with DAB solution for 10 min and sealed. Images were acquired using a confocal microscope.

### BMEC Isolation

Primary BMECs were isolated from 3–4-week-old rats based on a previous description [27]. Briefly, the cerebral cortex was dissected and digested with gentle trituration every 10 min for 30 min at 37 °C with PBS containing 10 mg/mL DNase I and 400 U/mL collagenase II. The resulting pellet was thoroughly washed with PBS over a 70-μm nylon mesh. After adequate centrifugation at 1000 rpm for 10 min, the cell pellets were resuspended in 25% BSA and centrifuged at 2000 rpm at 4 °C for 20 min. The cell pellets were resuspended in complete medium and seeded into plates coated with collagen (Abcam) at 37 °C in humidified 5% CO_2_/95% air. One day later, the culture medium was replaced by EC culture medium. After 10–12 days in culture, the cells were collected for further detections.

### Exposure of BMECs to the OGD/R Treatment

The sham group was incubated with high-glucose DMEM (HyClone) with 20% FBS at 37 °C in a 5% CO_2_/95% air atmosphere for 4 h. Then, the medium was replaced by a normal medium containing high-glucose DMEM, 20% FBS, 2 mmol/L glutamine, 100 units/mL penicillin, and 100 μg/mL streptomycin. BMECs were washed with PBS and incubated with glucose-free DMEM as an OGD medium. Then, the cells were transferred to an anaerobic chamber filled with a gas mixture of 1% O_2_, 95% N_2_, and 5% CO_2_ at 37 °C for 4 h as the OGD treatment (OGD group). After the OGD treatment, the cells were cultured in a normal medium for another 20 h for reoxygenation with or without the corresponding treatment containing 300 µg/ml rtPA (OGD + rtPA group) and/or 50 nM EX-4 (OGD + rtPA + EX-4 group) and/or 4 μM PRI-724 (OGD + rtPA + EX-4 + PRI-724 group) at 37 °C in a humidified atmosphere of 5% CO_2_/95% air.

### Cell Viability

The cells were seeded in 96-well plates (10,000 cells per well). After 20 h of treatments, the cell viability was determined using the CCK-8 assay kit (Beyotime Institute, Shanghai, China) in line with the manufacturer’s instructions.

### ROS Production Quantification

After the treatment, the sedimented cells were resuspended with PBS and incubated with DCFH-DA (10 mM, Molecular Probes) for 30 min at 37 °C to measure intracellular ROS generation. The image was captured by fluorescent microscopy immediately. The mean fluorescence intensity was evaluated by flow cytometry. The results were analyzed by using FlowJo 10 software.

### TUNEL Staining

Apoptotic BMECs were labeled with terminal deoxynucleotidyl transferase (TdT)–mediated dUTP nick end labeling (TUNEL) according to the manufacturer’s instructions (Beyotime Biotechnology, China). The number of positive BMECs was observed by fluorescence microscopy and counted in 8 microscopic fields under 200 × magnification.

### Immunofluorescence

The brains were cut into 25-μm-thick sections for immunostaining analysis. Based on our established protocol [28], the slices were blocked with PBS containing 5% BSA and incubated with 0.25% Triton-X 100. Next, the slices were incubated with primary antibodies, including rabbit anti-Factor VIII (1:200, Bioss) and rabbit anti-MMP-9 (1:200, Abcam), overnight at 4 °C, followed by incubation with the appropriate Alexa 488 or 594-conjugated secondary antibody (1:500, Jackson). The brain sections were washed with PBS and then incubated in DAPI. The brain sections were sealed with 50% glycerin and observed under a fluorescence microscope.

### Western Blot Analysis

Eight percent to 12% SDS-PAGE gels was electrophoresed with some amounts of protein per lane (20 μg) based on our established protocol [16, 29]. Proteins were electrophoretically transferred onto polyvinylidene difluoride (PVDF) membranes and blocked with 5% skimmed milk in TBST for 1 h at room temperature. Thereafter, the PVDF membranes were incubated with different primary antibodies, including rabbit anti-Occludin-1 (1:1000, Invitrogen), rabbit anti-MMP-9 (1:1000, Abcam), rabbit anti-β-catenin (1:2000, Abcam), rabbit anti-p-β-catenin (1:1000, Abcam), rabbit anti-GSK-3β (1:2000, Abcam), rabbit anti-Caspase-3 (1:2000, ABclonal), rabbit anti-GAPDH (1:3000, Abcam), mouse anti-Claudin-5 (1:1000, Invitrogen), and rat anti-ZO-1 (1:500, Santa Cruz Biotechnology). After washing with TBST, the PVDF membranes were treated with HRP-conjugated secondary antibody (1:5000) for 1 h at room temperature. Immunolabeling was probed with enhanced ECL kit (Biosharp). The chemiluminescence level can be captured with the imaging system, and the data were normalized to GAPDH.

### Statistical Analysis

All the data were analyzed by using the GraphPad Prism version 5.04 statistical package and were expressed as mean ± standard deviation (SD). Differences among the groups were determined by one-way analysis of variance (ANOVA). The differences were considered statistically significant at *p* < 0.05.

## Results

### Effect of EX-4 on Neurologic Deficit Score, Cerebral Edema, and Infarct Area in Rats After MCAO Treated with rtPA

**Fig. 1:**
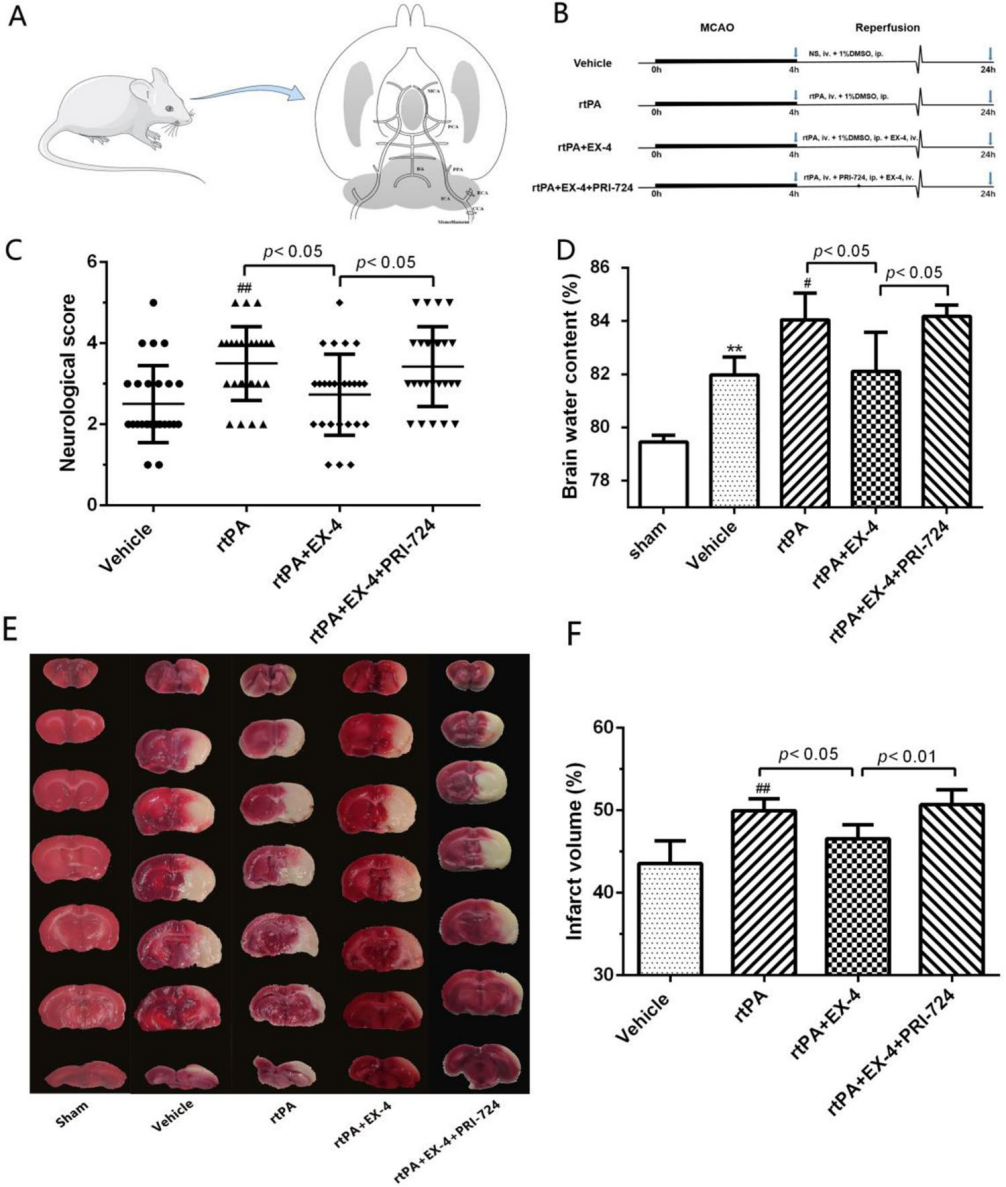
EX-4 reduces neurologic deficit and infarct volume in rats after MCAO treated with rtPA. **a** In vivo, MCAO was performed by monofilament in rats. **b** Schematic diagram of the experimental design. The experimental groups were treated with vehicle, rtPA, rtPA + EX-4, or rtPA + EX-4 + PRI-724. **c** Rat performance on the neurologic deficit scores at 24 h after MCAO is shown in bar graphs (*n* = 25 rats/group). **d** Bar graphs show the brain water content at 24 h after MCAO (*n* = 4 rats/group). **e** Coronal sections immersed with TTC at 24 h after MCAO show infarct area (unstained regions). **f** Bar graphs show infarct volumes of each group (*n* = 5 rats/group). Data are shown as mean ± SD and analyzed by one-way ANOVA. *p < 0.05 vs. sham group, **p < 0.01 vs. sham group; #*p* < 0.05 vs. vehicle group, ##*p* < 0.01 vs. vehicle group

The diagram of MCAO anatomy and the schematic diagram of the research design and experimental groups are displayed in Fig. 1a, b. The MCAO rats exerted significantly neurologic deficit by their behavior in the neurologic deficit score. rtPA administration worsened the neurologic performance compared with the vehicle group (*p* < 0.01; Fig. 1c). The MCAO rats treated with rtPA + EX-4 exerted significant improvement on the neurologic deficit score at 24 h after MCAO (*p* < 0.05). These rats treated with rtPA + EX-4 + PRI-724 performed worse than the MCAO rats treated with rtPA + EX-4 (*p* < 0.05). Cerebral edema measured by wet-to-dry weight was significantly higher in the vehicle group than in the sham group (*p* < 0.01; Fig. 1d). The MCAO rats treated with rtPA showed aggravated cerebral edema compared with the vehicle group (*p* < 0.05). The increase in rtPA-induced edema was inhibited by EX-4 administration (*p* < 0.05), and the MCAO rats treated with rtPA + EX-4 + PRI-724 had more pronounced cerebral edema compared with the rtPA + EX-4 group (*p* < 0.05) at 24 h after MCAO. The MCAO rats treated with rtPA showed the enlarged infarct compared with the vehicle group (*p* < 0.01; Fig. 1e, f). The combination treatment with rtPA and EX-4 decreased the infarct volume compared with the treatment with rtPA alone (*p* < 0.05). The MCAO rats treated with rtPA + EX-4 + PRI-724 had a larger infarct volume than the rtPA + EX-4 group (*p* < 0.01) at 24 h after MCAO.

### Effect of EX-4 on HT and BBB Permeability After MCAO Treated with rtPA

**Fig. 2:**
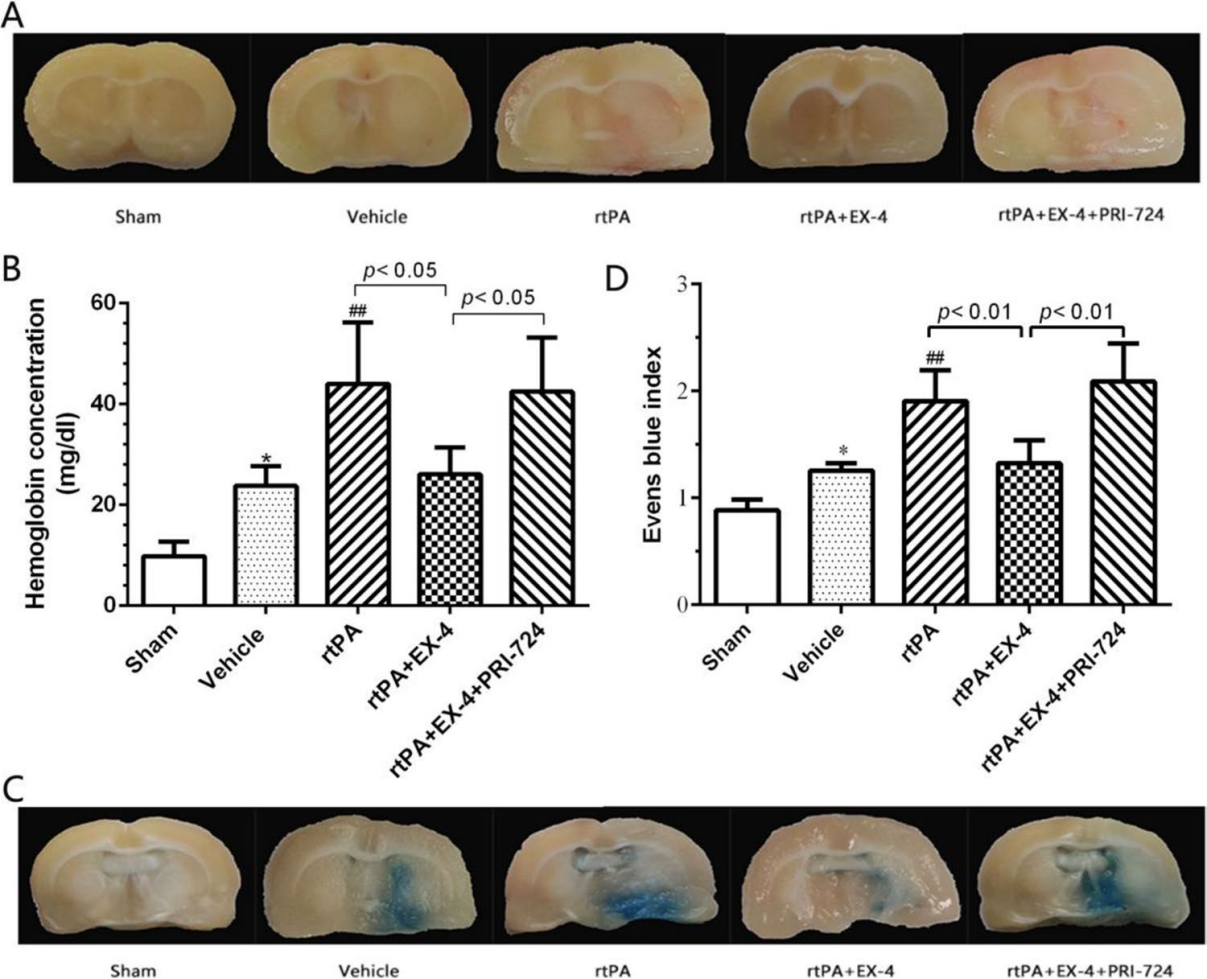
EX-4 reduces rtPA-induced hemorrhagic transformation and BBB permeability after cerebral ischemia. **a** Coronal section shows the level of hemorrhage 24 h after MCAO. **b** Bar graphs show the quantification of hemorrhage volume measured by hemoglobin spectrophotometric assay (*n* = 5 rats/group). **c** Bar graphs show the content of the EB in the brain (*n* = 5 rats/group). **d** Coronal section shows the extravasation of the EB dye 24 h after MCAO. Data are shown as mean ± SD and analyzed by one-way ANOVA. **p < 0.01 vs. sham group; ##*p* < 0.01 vs. vehicle group

All the coronal sections were observed; we found that the hemorrhage was not significant in the vehicle group and rtPA + EX-4 group. In contrast, there was increased hemorrhage in the rtPA group and rtPA + EX-4 + PRI-724 group 24 h after MCAO (Fig. 2a). The hemoglobin concentration of the ipsilateral hemisphere was measured by hemoglobin spectrophotometric assay. Significantly increased hemorrhage reduced by rtPA was inhibited by EX-4 treatment 24 h after MCAO (*p* < 0.05; Fig. 2b). Moreover, the degree of EB dye extravasation was observed to examine the BBB permeability in each group (Fig. 2c). Namely, the treatment with rtPA alone significantly aggravated EB dye extravasation compared with the vehicle group (*p* < 0.01; Fig. 2c), whereas MCAO rats treated with rtPA + EX-4 had lower EB dye extravasation compared with the rtPA group (*p* < 0.05; Fig. 2d) at 24 h after MCAO. Moreover, the rtPA + EX-4 + PRI-724 treatment increased the hemorrhage and the EB dye extravasation compared with the rtPA + EX-4 treatment. These data showed the effect of EX-4 on protecting the BBB integrity and implicated that the Wnt/β-catenin signaling pathway may participate in the rtPA-related HT and BBB damage.

### Effect of EX-4 on the Disruption of the BBB After MCAO Treated with rtPA

**Fig. 3:**
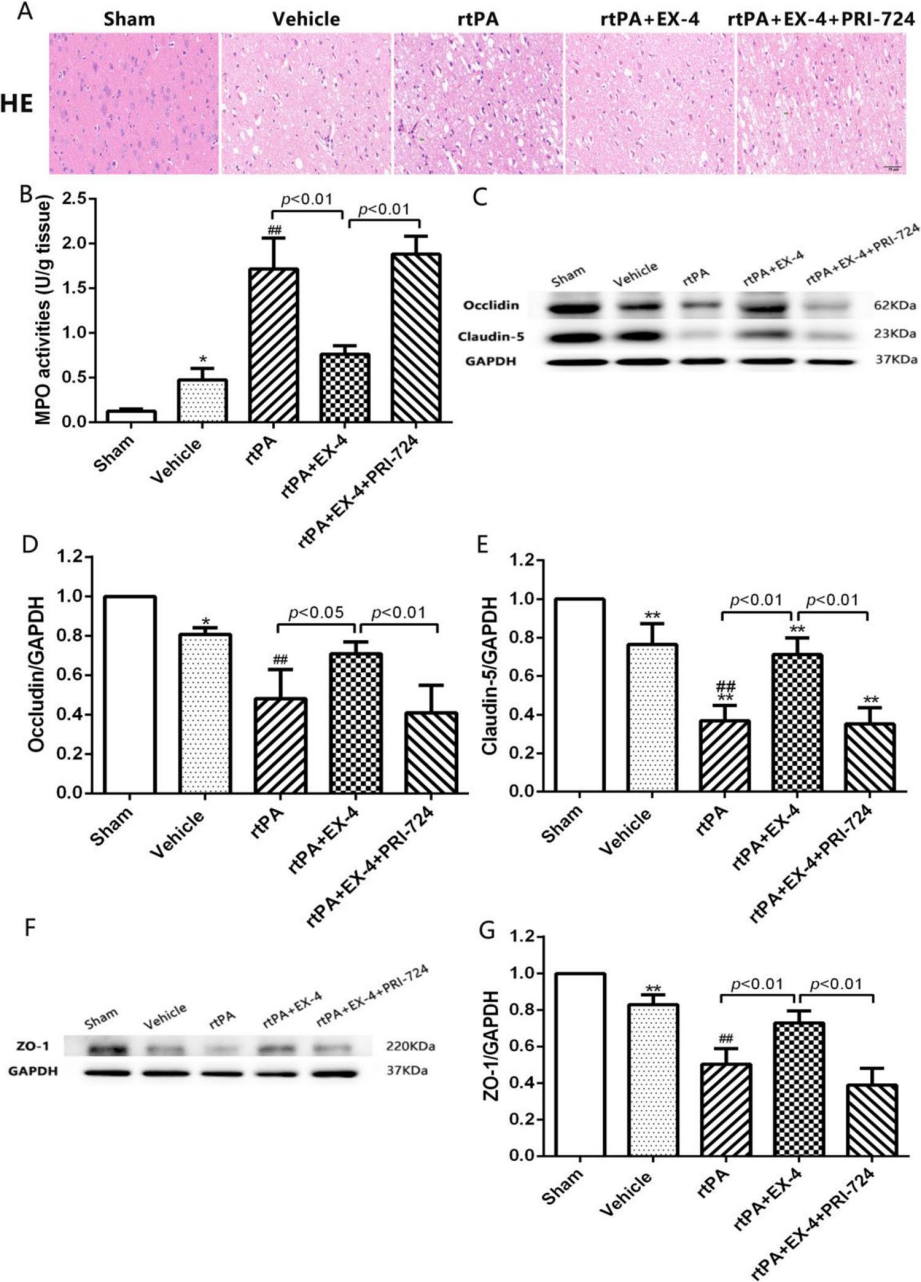
EX-4 reduces rtPA-induced BBB damage after cerebral ischemia. **a** Histopathological changes tested by HE staining in infarcted cortex (original magnification × 400). **b** The MPO activity was detected in the infarcted lateral hemisphere (*n* = 4 rats/group). **c–e** Immunoblot images and quantitative data of the Occludin and Claudin-5 at 24 h after MCAO (*n* = 4 rats/group). **f, g** Immunoblot images and quantitative data of the ZO-1 at 24 h after MCAO (*n* = 4 rats/group). Data are shown as mean ± SD and analyzed by one-way ANOVA. *p < 0.05 vs. sham group, **p < 0.01 vs. sham group, ##*p* < 0.01 vs. vehicle group

In this study, HE staining was used to assess the histological changes in the cerebral cortex. The normal brain structure showed integral histological and cellular structure. While the cerebral structure in the vehicle group revealed edema and few inflammatory cells, the rtPA group showed increased edema, inflammatory cells, and hemorrhage (Fig. 3a). The treatment with EX-4 ameliorated rtPA-induced histological changes compared with the rtPA group 24 h after MCAO. Moreover, the usage of PRI-724 aggravated the histological injury compared with the rtPA + EX-4 + PRI-724 group. The MPO activity of the brain tissue, reflecting neutrophil infiltration, significantly increased in the rtPA group compared with the vehicle group (*p* < 0.01; Fig. 3b). The MCAO rats treated with rtPA + EX-4 showed a decreased MPO activity compared with the rtPA group (*p* < 0.01), whereas the rtPA + EX-4 + PRI-724 group reversed this protective effect at 24 h after MCAO (*p* < 0.01).

Western blotting showed that the band density of ZO-1, Occludin, and Claudin-5 was reduced in the vehicle group at 24 h after MCAO. The treatment with rtPA aggravated the degradation of TJPs compared with the vehicle group (*p* < 0.01; Fig. 3c–g). EX-4 significantly suppressed the rtPA-induced disruption of ZO-1, occludin, and Claudin-5, but PRI-724 reversed these effects 24 h after MCAO.

### Effect of the Wnt/β-Catenin Signaling Pathway on rtPA-Associated HT at 24 h After MCAO in Rats

**Fig. 4:**
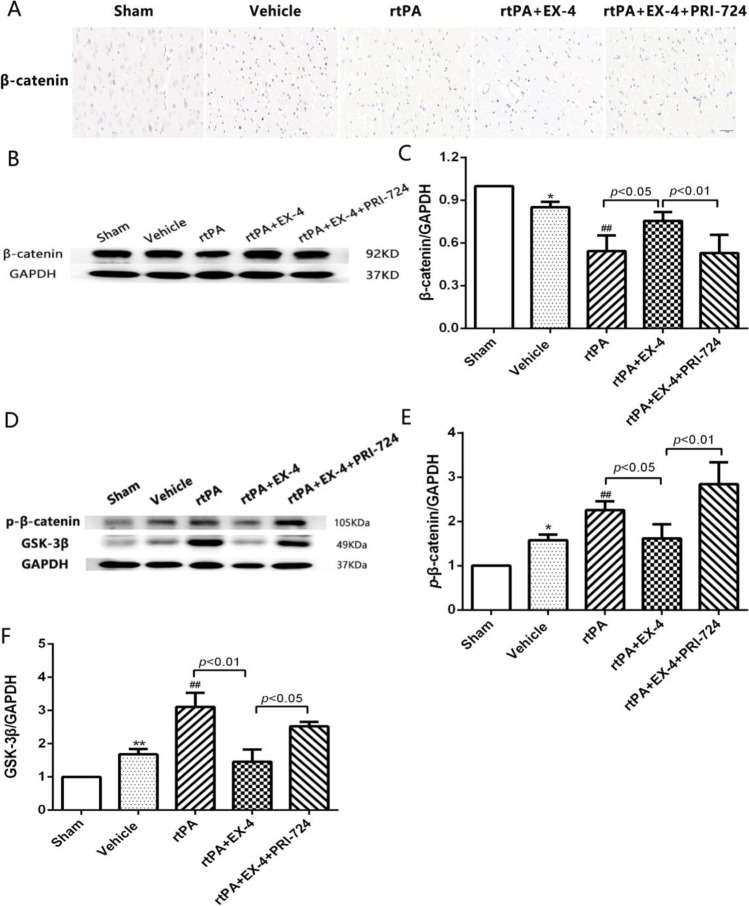
Wnt/β-catenin signaling pathway plays an important role in rtPA-associated HT after MCAO in rats. **a** Representative images of β-catenin expression detected by immunohistochemical staining in the infarcted cortex (original magnification × 400). **b, c** Immunoblot images and quantitative data of β-catenin expression at 24 h after MCAO (*n* = 4 rats/group). **d–f** Immunoblot images and quantitative data of protein level of p-β-catenin and GSK-3β at 24 h after MCAO (*n* = 4 rats/group). Data are shown as mean ± SD and analyzed by one-way ANOVA. *p < 0.05 vs. sham group, **p < 0.01 vs. sham group, and ##*p* < 0.01 vs. vehicle group

Immunohistochemistry was used to assess the expression level of β-catenin in the brain tissue (Fig. 4a). The expression level of β-catenin was significantly inhibited in the rtPA group compared with the vehicle group 24 h after MCAO. The treatment with rtPA + EX-4 ameliorated rtPA-induced downregulation of β-catenin at 24 h after MCAO, but PRI-724 decreased the β-catenin expression. We also explored the expression of p-β-catenin and GSK-3β in the MCAO rats after rtPA thrombolysis (Fig. 4b–f). Western blotting revealed a significant upregulation of p-β-catenin and GSK-3β in the rtPA group compared with the vehicle group at 24 h after MCAO (Fig. 4d–f). EX-4 significantly suppressed the rtPA-induced expression of p-β-catenin (*p* < 0.05) and GSK-3β (*p* < 0.01), whereas PRI-724 reversed this effect 24 h after MCAO, suggesting that the activation of the Wnt/β-catenin signaling pathway may reduce rtPA-induced HT 24 h after MCAO in rats.

### Effect of Wnt/β-Catenin Signaling Pathway on MMP-9 Activation in rtPA-Associated HT at 24 h After MCAO in Rats

**Fig. 5:**
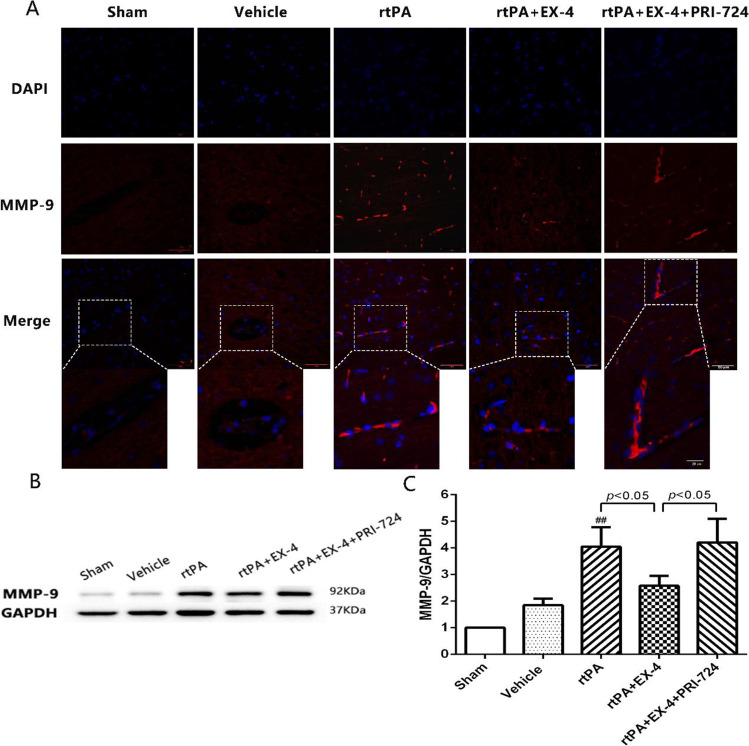
EX-4 reduces the expression of MMP-9 in rtPA-associated HT after MCAO in rats. **a** Representative images of MMP-9 expression detected by immunofluorescence in the infarcted cortex (original magnification × 400). **b, c** Immunoblot images and quantitative data of the expression of MMP-9 at 24 h after MCAO (*n* = 4 rats/group). Data are shown as mean ± SD and analyzed by one-way ANOVA. *p < 0.05 vs. sham group, **p < 0.01 vs. sham group, and ##*p* < 0.01 vs. vehicle group

To further analyze the mechanism of rtPA-induced BBB damage after MCAO, we detected the expression levels of MMP-9 by immunofluorescence in the ipsilateral brain tissue (Fig. 5a). We found that the expression of MMP-9 was obvious in cerebral microvessels in the rtPA group. EX-4 reduced the activation of MMP-9, while PRI-724 upregulated MMP-9 activation, especially in cerebral microvessels. Western blotting showed increased MMP-9 expression in the vehicle group compared to the sham group, and expression of MMP-9 in the rtPA group was significantly increased compared to the vehicle group (Fig. 5b, c). rtPA-induced MMP-9 expression was inhibited by EX-4, but the effect was reversed by PRI-724, indicating that the Wnt/β-catenin signaling pathway can participate in rtPA-induced MMP-9 activation 24 h after MCAO in rats.

### Effect of the Wnt/β-Catenin Signaling Pathway *In Vitro* on BMECs in the OGD/R Model Treated with rtPA

**Fig. 6:**
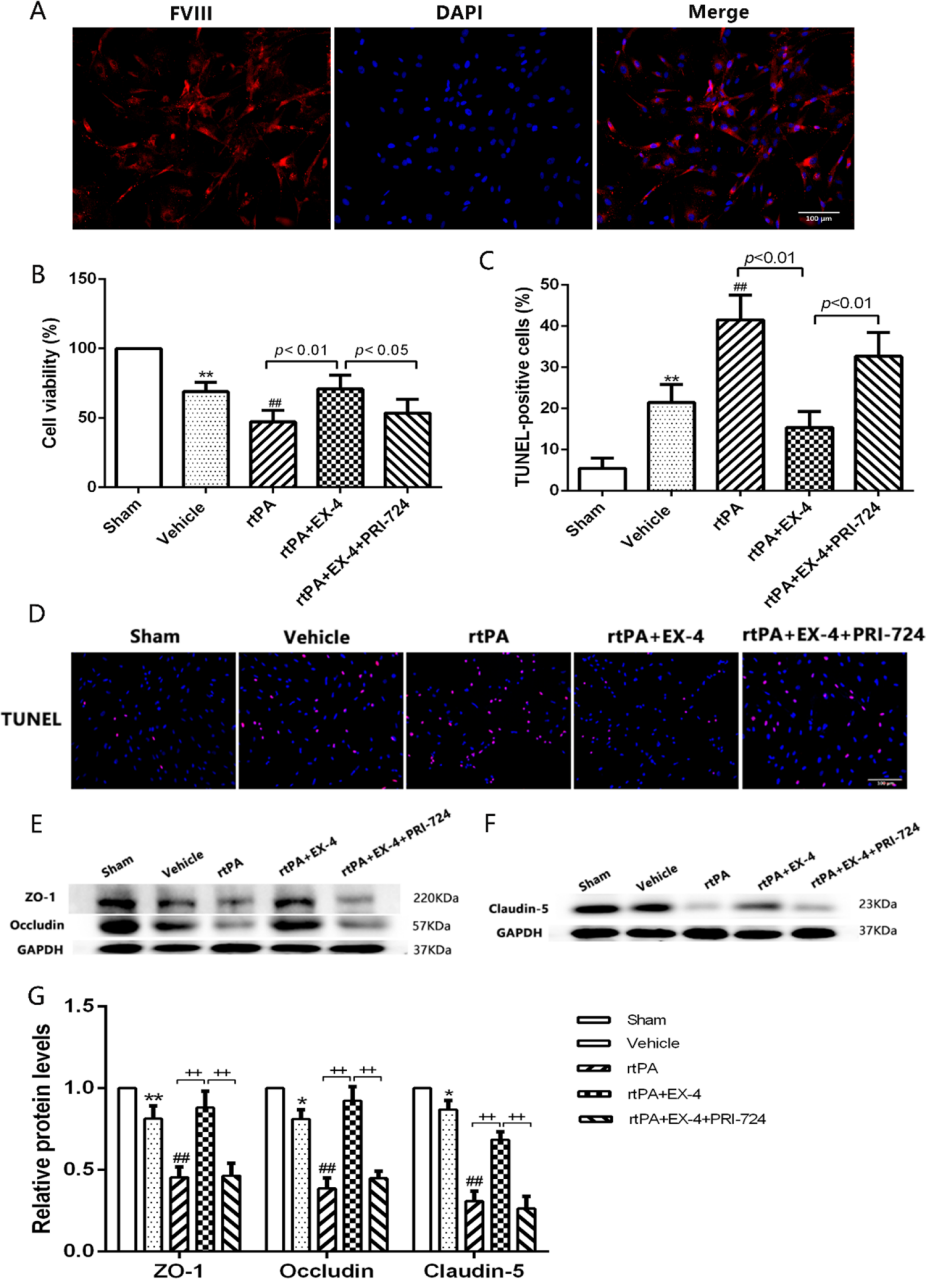
Effect of EX-4 on protecting the BMEC viability and reducing the tPA-induced tight-junction protein damage in the OGD/R model in vitro. (**A**) The purity of primary BMECs was detected by immunofluorescence images (original magnification × 200). (**B**) Viability of the BMECs treated by different treatments was accessed by the CCK-8 kit in the OGD/R model in vitro (*n* = 5 in each group). **c, d** Apoptosis of BMECs treated by different treatments was tested by TUNEL assay in the OGD/R model in vitro (original magnification × 200). **e–g** Immunoblot images and quantitative data of the expression of the ZO-1, Occludin, and Claudin-5 in BMECs in the OGD/R model in vitro (*n* = 4 in each group). Data are shown as mean ± SD and analyzed by one-way ANOVA. *p < 0.05 vs. sham group, **p < 0.01 vs. sham group, ##*p* < 0.01 vs. vehicle group. + *p* < 0.05 between two groups and +  + *p* < 0.01 between two groups

BMECs are important components of the BBB, and they exert a crucial effect on the occurrence of HT. To further analyze the degree of rtPA-induced BMECs damage after OGD/R, primary BMECs were isolated from SD rats and the cell purity was greater than 95%, which detected by immunofluorescence FVIII labeling of endothelial cells (Fig. 6a). We also evaluated cell viability of each group and TUNEL staining (Fig. 6b–d). The treatment of BMECs with rtPA significantly decreased the cell viability and promoted apoptosis at 24 h after OGD/R (*p* < 0.01). EX-4 improved the viability of BMECs compared with the rtPA group (*p* < 0.01) and reduced the rtPA-induced apoptosis of BMECs (*p* < 0.01). PRI-724 aggravated the BMECs damage and cell apoptosis 24 h after OGD/R.

Furthermore, we explored the expression of tight-junction proteins in BMECs 24 h after OGD/R. Western blotting showed that the band density of ZO-1, Occludin, and Claudin-5 was reduced in the vehicle group at 24 h after OGD/R (Fig. 6e–g). rtPA deteriorated the disruption of ZO-1, Occludin, and Claudin-5 compared with the vehicle group (*p* < 0.01). EX-4 significantly inhibited the rtPA-induced damage of ZO-1, Occludin, and Claudin-5, whereas PRI-724 reversed this effect at 24 h after OGD/R. These results showed that activating the Wnt/β-catenin signaling pathway may decrease rtPA-induced BMECs damage and tight-junction protein damage.

### Effect of the Wnt/β-Catenin Signaling Pathway on rtPA-Induced BMEC Damage at 24 h After OGD/R

**Fig. 7:**
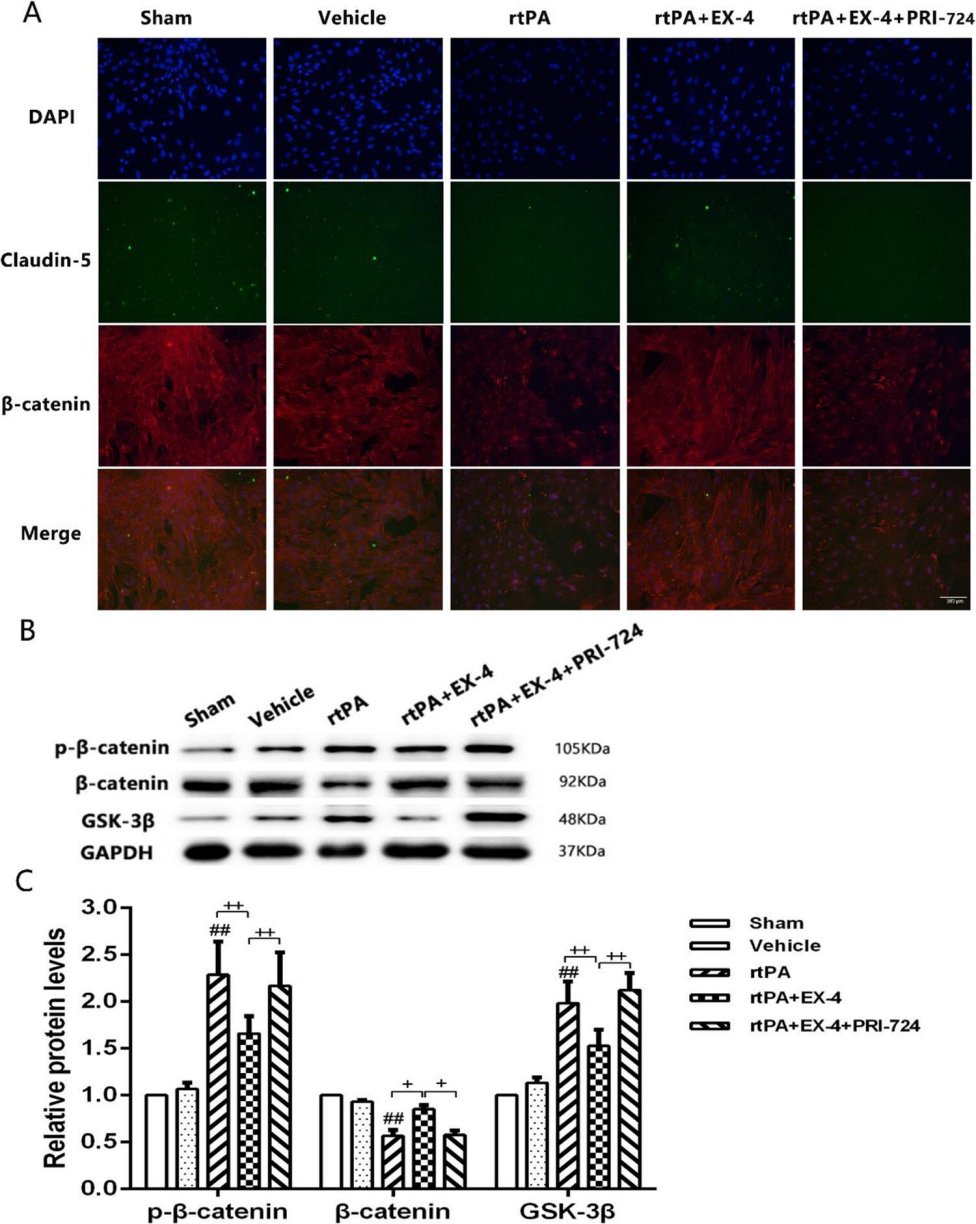
Wnt/β-catenin signaling pathway plays an important role in the BMECs rtPA-induced OGD/R model in vitro. **a** Representative images of β-catenin and Claudin-5 expression detected by immunofluorescence (original magnification × 200). **b, c** Immunoblot images and quantitative data of the expression of p-β-catenin, β-catenin, and GSK-3β 24 h after MCAO (*n* = 4 in each group). Data are shown as mean ± SD and analyzed by one-way ANOVA. **p < 0.01 vs. sham group; ##*p* < 0.01 vs. vehicle group. + *p* < 0.05 between two groups, +  + *p* < 0.01 between two groups

Immunofluorescence was used to detect the expression of β-catenin in BMECs (Fig. 7a). The expression of β-catenin was significantly inhibited in the rtPA group compared with the vehicle group 24 h after OGD/R. The rtPA + EX-4 treatment ameliorated the rtPA-induced downregulation of β-catenin compared with the rtPA treatment alone, but PRI-724 reduced the expression of β-catenin at 24 h after OGD/R. We also explored the expression level of β-catenin, p-β-catenin, and GSK-3β by western blotting in the BMEC OGD/R model. The results showed a significant upregulation of p-β-catenin and GSK-3β and downregulation of β-catenin in the rtPA group compared with the vehicle group at 24 h after OGD/R (*p* < 0.01; Fig. 7b, c). EX-4 significantly suppressed the rtPA-induced expression of p-β-catenin, and GSK-3β and increased the expression of β-catenin, whereas PRI-724 reversed this effect at 24 h after OGD/R. These findings indicated that the Wnt/β-catenin signaling pathway plays an important role in rtPA-induced BMEC damage, and the activation of this pathway may diminish the BMEC damage.

### Effect of the Wnt/β-Catenin Signaling Pathway on MMP-9 Activation in rtPA-Associated BMEC Damage at 24 h After OGD/R

**Fig. 8:**
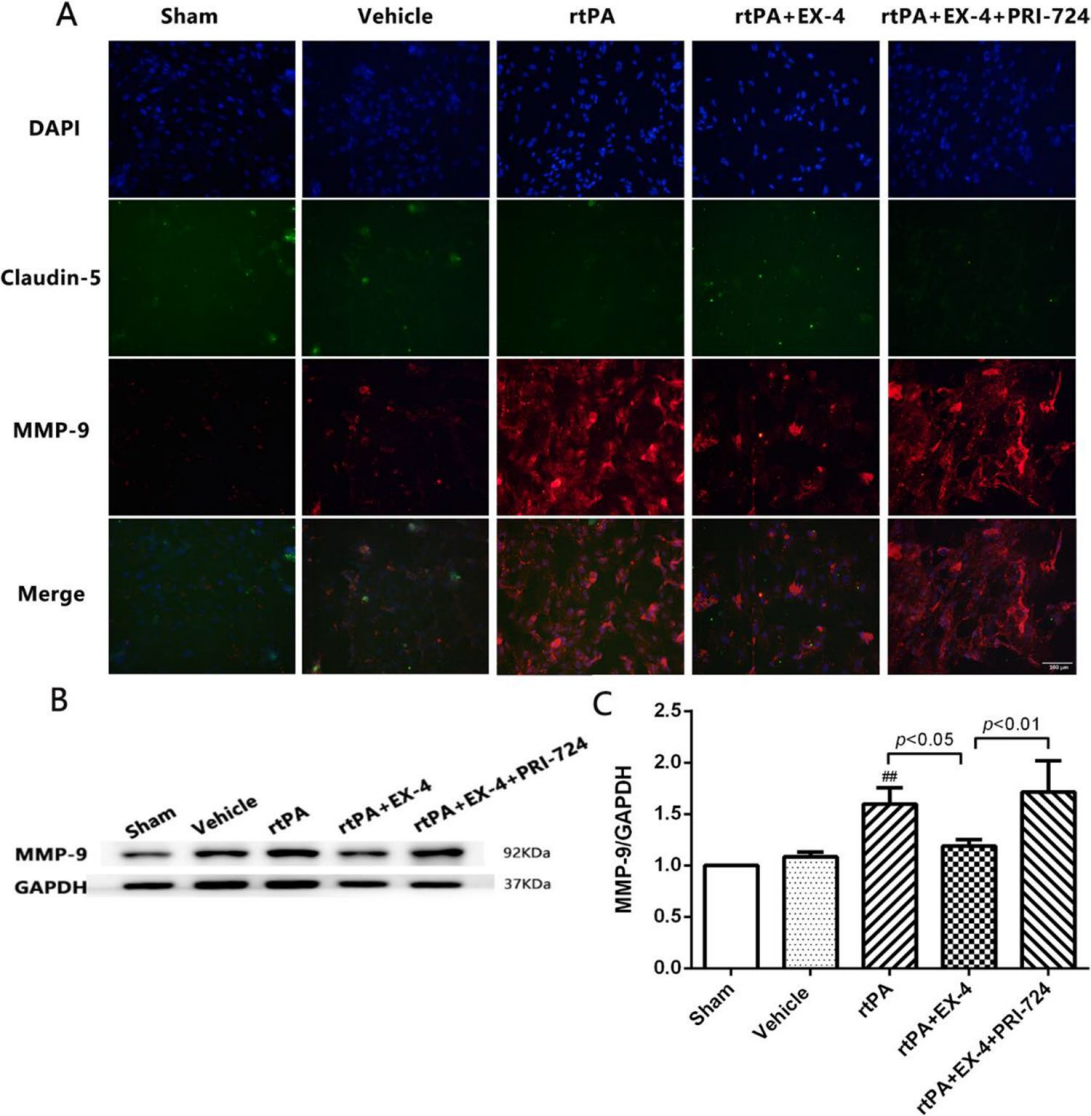
EX-4 reduces rtPA-associated MMP-9 activation in the BMECs OGD/R model in vitro. **a** Representative images of MMP-9 and Claudin-5 expression detected by immunofluorescence in the BMECs OGD/R model in vitro (original magnification × 200). **b, c** Immunoblot images and quantitative data of the expression of MMP-9 in the BMECs OGD/R model in vitro (*n* = 4 in each group). Data are shown as mean ± SD and analyzed by one-way ANOVA. **p < 0.01 vs. sham group; ##*p* < 0.01 vs. vehicle group

To further analyze the mechanism of rtPA-induced BMECs damage at 24 h after OGD/R, we detected the expression levels of MMP-9 and Claudin-5 by immunofluorescence in BMECs in vitro (Fig. 8a). We found that the expression of MMP-9 was significantly higher in the rtPA group than in the vehicle group. EX-4 was able to reduce the activation of MMP-9, but PRI-724 upregulated the expression levels of MMP-9 at 24 h after OGD/R. Western blotting was used to measure the expression levels of MMP-9 (Fig. 8b, c). We also found that rtPA significantly induced the MMP-9 expression of the BMECs at 24 h after OGD/R compared with the vehicle. EX-4 reduced the expression of MMP-9 by activating the Wnt/β-catenin pathway (*p* < 0.05), which could be reversed by PRI-724 (*p* < 0.01). These findings suggested that the Wnt/β-catenin signaling pathway may affect rtPA-induced BMECs damage by regulating MMP-9 expression at 24 h after OGD/R.

### Effect of the Wnt/β-Catenin Signaling Pathway on ROS Production in rtPA-Induced BMEC Damage at 24 h After OGD/R

**Fig. 9:**
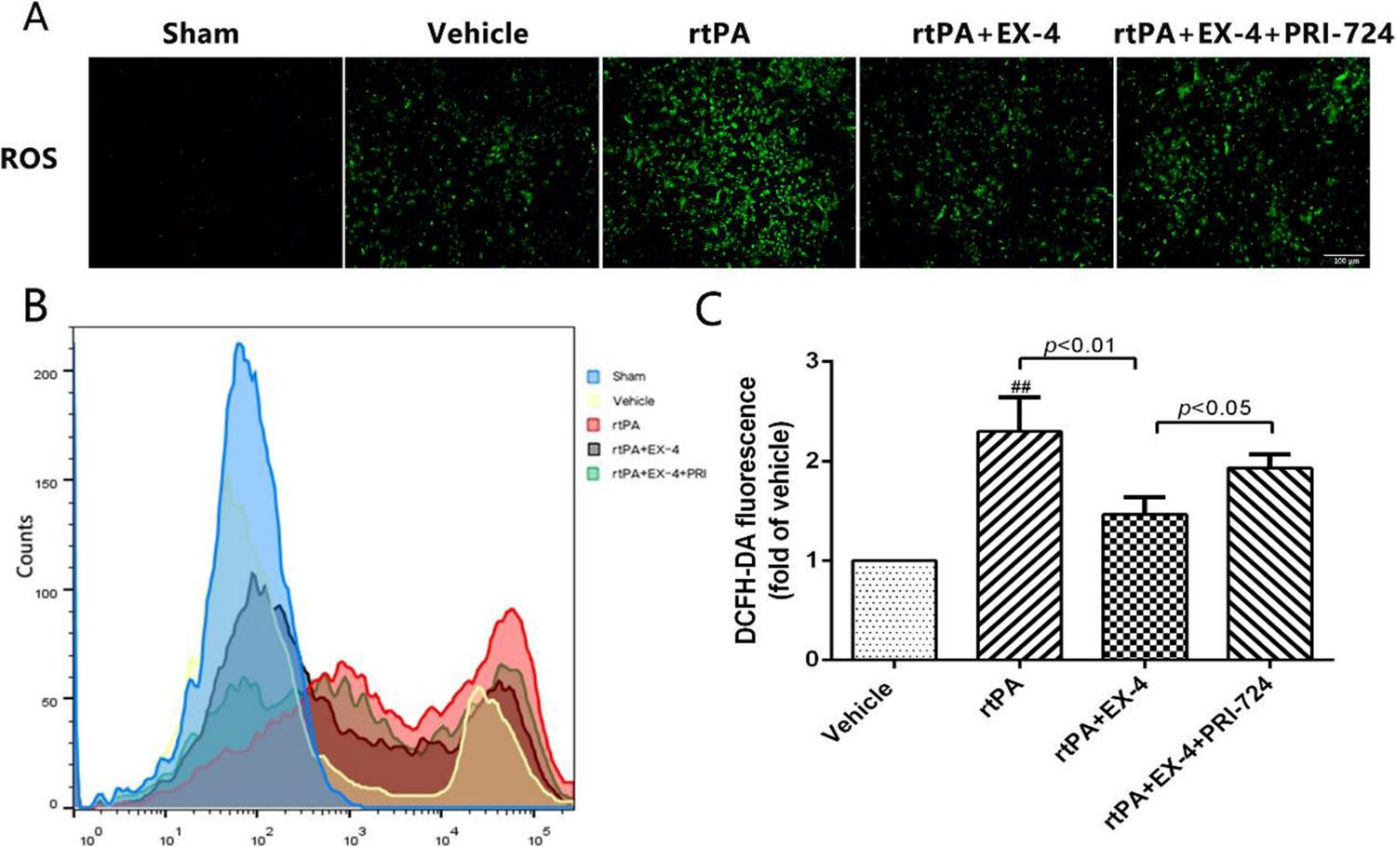
EX-4 reduces rtPA-associated ROS production in BMECs 24 h after OGD/R in vitro. **a** Representative images of ROS expression detected by immunofluorescence in BMECs 24 h after OGD/R in vitro (original magnification × 200). **b, c** Flow cytometry analysis of ROS in BMECs 24 h after OGD/R in vitro. Bar graphs illustrate the quantitative analysis (*n* = 4 in each group). Data are shown as mean ± SD and analyzed by one-way ANOVA. **p < 0.01 vs. sham group; ##*p* < 0.01 vs. vehicle group

Since oxidative factors play a crucial role in cerebral ischemia [30], we further analyzed the mechanism of rtPA-induced BMECs damage at 24 h after OGD/R, and we assessed the levels of ROS by immunofluorescence in BMECs in vitro (Fig. 9a). We found that the levels of ROS were significantly increased in the vehicle group compared with the sham group, and the levels of ROS increased more in the rtPA group than in the vehicle group. EX-4 reduced the production of ROS, but PRI-724 reversed the protection of EX-4 in BMECs in vitro at 24 h after OGD/R. Our results showed significantly increased levels of ROS in the rtPA group compared with the vehicle group (*p* < 0.01; Fig. 9b, c). The rtPA-induced ROS production was diminished by inhibiting the Wnt/β-catenin pathway under the action of EX-4, effect of which could be inhibited by PRI-724. These results implicated that rtPA-induced ROS production can be influenced by the Wnt/β-catenin signaling pathway in BMECs in vitro at 24 h after OGD/R.

## Discussion

**Fig. 10:**
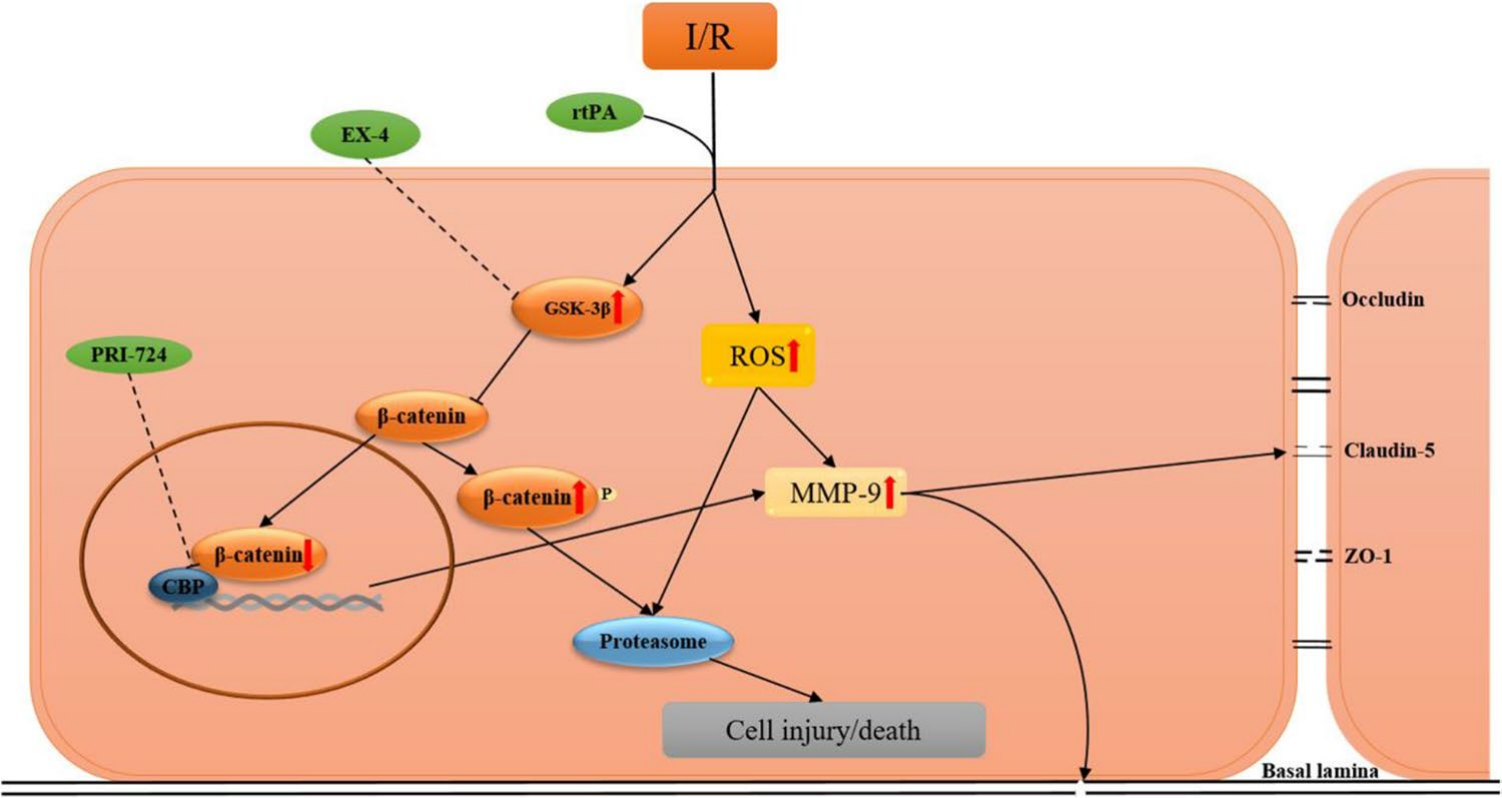
A summary diagram to show the mechanisms of HT after ischemic stroke treated by rtPA via Wnt/β-catenin signaling pathway. I/R: ischemic/reperfusion. rtPA: tissue-type plasminogen activator. ROS: reactive oxygen species. MMP-9: matrix metalloproteinase-9. GSK-3β: glycogen synthase kinase-3β. ZO-1: zonula occludens-1

Recently, many different drugs acting on various mechanisms have been developed to intervene at various steps of ischemia–reperfusion injury or BBB damage, so as to decrease the incidence of HT after AIS. In this study, the risk of HT after rtPA thrombolysis and the effect of EX-4 on a rat MCAO model and on BMECs in vitro were observed. EX-4 played a protective role in reducing neurological deficits, brain edema, infarct volume, neutrophil infiltration, BBB damage, and hemorrhage in the MCAO model treated with rtPA. EX-4 protected the viability of BMECs from rtPA treatment in OGD/R model in vitro. In addition, EX-4 reduced degradation of tight-junction proteins by inhibiting MMP-9 activation and ROS production via activating Wnt/β-catenin signaling pathway. However, PRI-724, a selective inhibitor of β-catenin [31], was able to reverse the therapeutic effect of EX-4 in both the MCAO model and the OGD/R model. Our results indicated that the activation of the Wnt/β-catenin signaling pathway could be a potential strategy to reduce the risk of HT after ischemic stroke treated with rtPA, and EX-4 might be a potential therapeutic drug to diminish the adverse effect of HT by activating the Wnt/β-catenin signaling pathway. Moreover, the activation of the Wnt/β-catenin signaling pathway may inhibit MMP-9 activation and ROS production. The potential mechanisms of HT after rtPA thrombolysis in BMECs via the Wnt/β-catenin signaling pathway are shown in Fig. 10.

GLP-1 is a hormone that promotes insulin release in hyperglycemic conditions [32]. EX-4 mediates numerous physiological pathways through the G-protein-coupled GLP-1 receptor which is widely expressed in a variety of tissues, especially in the brain [33, 34]. The activation of GLP-1R exerts neuroprotection in some animal models of Alzheimer’s diseases, Parkinson’s diseases, and stroke [33, 35]. It has been reported that EX-4 could exert anti-inflammatory and protective effects on the BBB and neurons by reducing oxidative stress, which may contribute to the treatment of stroke [11, 12]. However, the role of EX-4 in HT of stroke after tPA treatment is still unclear. Unlike previous research reports, our results showed that the effect of rtPA combined with EX-4 may play a protective role on HT after ischemic stroke by regulating the Wnt/β-catenin signaling pathway.

Previous studies showed that the activation of the Wnt/β-catenin pathway may protect the BBB and reduce the risk of HT in AIS [36–38]. However, the mechanisms of the protective effect of the Wnt/β-catenin pathway activation against rtPA-aggravated HT remain unknown. MMP-9 is a zinc- and calcium-dependent proteolytic enzyme that can participate in the degradation of the BBB in rtPA-associated hemorrhagic complications after stroke [39, 40]. The activated MMP-9 can disrupt basal lamina and TJPs to damage BBB integrity in the experimental MCAO models [40]. In the vehicle group, the expression of MMP-9 was slightly increased, while in the rtPA group, the expression of MMP-9 was significantly increased, indicating that MMP-9 plays an important role in rtPA-induced BBB destruction. In the immunofluorescence images of rat brain tissue, we found that the expression of MMP-9 in the cerebral microvascular endothelial cells was significantly increased after rtPA treatment.. Since the cerebral microvascular endothelial cells are an important part of the BBB, we isolated the primary BMECs for further study. In addition, a high concentration of ROS induced by cerebral ischemia–reperfusion can activate MMP-9 to aggravate the BBB damage and lead to cell death by lipid peroxidation, protein dysfunction, and DNA damage [41]. Our experiments showed that rtPA significantly reduced the activation of MMP-9 and the production of ROS in the rat MCAO model and in BMECs OGD/R model in vitro. EX-4 was previously shown to inhibit MMP-9 activation and reduce infarct growth after focal cerebral ischemia in hyperglycemic mice [42]. Our results further suggested that EX-4 can suppress the activation of MMP-9 in ischemic brain tissue, especially in BMECs. It has been implied that EX-4 can alleviate ischemia–reperfusion injury by suppressing oxidative stress and inflammatory reaction in the mouse MCAO model [12, 14, 43, 44]. In addition, EX-4 was shown to attenuate neuroinflammation and BBB disruption through the PI3K/Akt pathway in warfarin-associated HT after cerebral ischemia [45]. In this study, GLP-1R agonist EX-4 inhibited MMP-9 activation, which may be due to the decrease in ROS level and reduction in the infiltration of leukocytes [46]. The Wnt/β-catenin pathway activated by EX-4 may suppress neuroinflammation by limiting the infiltration of leukocytes which are major sources of MMP-9 [47]. Decreased ROS level may reduce cell damage and the destruction of TJP, including ZO-1, occludin and claudin-5.

Previous studies have shown possible mechanisms of HT with various treatment targets including MMP inhibition, ROS reduction, BMEC protection, and inflammation regulation [48–52]. The protection of the BBB plays an important role in decreasing the risk of HT after the rtPA treatment. The Wnt/β-catenin signaling pathway can participate in regulating the induction and maintenance of BBB characteristics during embryonic and postnatal development [5, 13]. GSK-3β, an evolutionarily conserved serine/threonine protein kinase, can affect some transcription factors and signaling proteins and regulate the phosphorylation and degradation of β-catenin [53]. GSK-3β may regulate the Nrf2/ARE pathway and decrease oxidative stress in cerebral ischemia–reperfusion [45]. GSK-3β inhibition can reduce the BBB damage and early ischemia/reperfusion injury in stroke [15, 54]. TWS119, a GSK-3β inhibitor, attenuates rtPA-induced HT by activating the Wnt/β-catenin pathway after AIS in rats [16]. Here, we discovered that EX-4 reduced GSK-3β expression to inhibit the degradation of β-catenin and activated the Wnt/β-catenin pathway. β-Catenin, a co-transcriptional factor, is the key factor of the Wnt/β-catenin pathway to form the APC-β-catenin complex and β-catenin-TCF complex for regulating the downstream signaling pathway [55]. PRI-724 can suppress the Wnt/β-catenin signaling pathway by restraining the interaction between β-catenin and CBP to inhibit the downstream effector pathways of β-catenin for reversing the protective effect of EX-4. With PRI-724 inhibiting combination with CBP and β-catenin, the accumulation of β-catenin facilitated rapid GSK-3β phosphorylation, which, in turn, promoted the β-catenin phosphorylation degradation process. This result further indicated that EX-4 can protect the integrity of the BBB via the Wnt/β-catenin pathway. The activation of the Wnt/β-catenin pathway may inhibit leukocyte infiltration and reduce rtPA-induced MMP-9 expression and ROS production, thereby reducing BBB tight-junction protein degradation and improving neurological outcomes. These findings have important clinical implications and may lead to new therapies for patients receiving rtPA thrombolytic therapy after AIS.

There are some limitations in our article. First, we used corresponding drugs to assess the effects of GLP-1R without knocking out or overexpressing the GLP-1R gene in vivo and in vitro. Second, the component of the BBB is complex. We studied the effects of EX-4 mainly on microvascular endothelial cells and intercellular junction proteins. Whether there are corresponding effects on astrocytes or pericyte deserves further study. Finally, the mechanism of the BBB disruption is very comprehensive in HT; microglial activation and inflammation also require further research.

Our study showed the effect of GLP-1R agonists on BBB in HT. Treatment with Ex-4 immediately is effective to decrease the occurrence of HT for patients receiving rtPA thrombolytic therapy after AIS. Clinical trials of rtPA in combination with GLP-1R agonists are needed to reduce the adverse effects of rtPA and protect the prognosis of patients.

## Conclusions

Our study indicated that the GLP-1 agonist protected the brain against HT and alleviated the rtPA-induced BBB disruption in ischemic stroke through the Wnt/β-catenin signaling pathway. Our results showed that the activation of the Wnt/β-catenin signaling pathway provided a protective effect, which was reflected in reduced ROS levels and leukocyte infiltration, thereby inhibiting the rtPA-induced MMP-9 activity and tight-junction protein degradation in the rat MCAO model and in the BMEC OGD/R model in vitro. These findings have significant clinical implications and could potentially be developed into novel therapies for patients receiving thrombolytic therapy after AIS. Our results suggested that the GLP-1 agonist may be a potential strategy to reduce ischemia–reperfusion injury and rtPA-induced HT via the Wnt/β-catenin signaling pathway.

## Data Availability

The datasets supporting the conclusions of this article are included within the article and its additional files. All material used in this manuscript will be made available to researchers subject to confidentiality.

## Abbreviations

tPA: Tissue plasminogen activator
AIS: Acute ischemic stroke
HT: Hemorrhagic transformation
rtPA: Recombinant tissue plasminogen activator
BBB: Blood–brain barrier
EX-4: Exendin-4
MCAO: Middle cerebral artery occlusion
OGD/R: Oxygen-glucose deprivation plus reoxygenation
BMECs: Brain microvascular endothelial cells
MMPs: Matrix metalloproteinases
ROS: Reactive oxygen species
GLP-1R: Glucagon-like peptide-1 receptor
GSK-3β: Glycogen synthase kinase-3β
SD: Sprague–Dawley
TTC: 2,3,5-Triphe-nyltetrazolium chloride
EB: Evans blue
MPO: Measurement of myeloperoxidase
HE: Hematoxylin–eosin
PVDF: Polyvinylidene difluoride
I/R: Ischemic/reperfusion
MMP-9: Matrix metalloproteinase-9
ZO-1: Zonula occludens-1
